# Satellite-based vertical land motion for infrastructure monitoring: a prototype roadmap in Greater Houston, Texas

**DOI:** 10.1038/s41598-025-01970-8

**Published:** 2025-05-16

**Authors:** B. Buzzanga, M. Govorcin, F. Kremer, J. E. Schubert, D. P. S. Bekaert, B. Schaeffer, P. Milillo, A. J. Williams, B. F. Sanders, A. L. Handwerger, S. Staniewicz

**Affiliations:** 1 Jet Propulsion Laboratory, California Institute of Technology, Pasadena, CA USA; 2 Joint Institute for Regional Earth System Science and Engineering, University of California Los Angeles, Los Angeles, CA USA; 3 U.S. Environmental Protection Agency, Office of Research and Development, Cincinnati, OH USA; 4 U.S. Environmental Protection Agency, Office of Research and Development, Research Triangle Park, Raleigh, NC USA; 5 Department of Civil and Environmental Engineering, University of Houston, Houston, TX USA; 6 Department of Earth and Atmospheric Science, University of Houston, Houston, TX USA; 7 Microwaves and Radar Institute, German Aerospace Center (DLR), Oberfaffenhofen, Germany; 8 Department of Civil and Environmental Engineering, University of California, Irvine, CA USA

**Keywords:** Natural hazards, Climate change

## Abstract

Coastal regions are critical hubs for industries reliant on transport and storage. However, vital infrastructure including above-ground storage tanks (ASTs), which store hazardous materials, is vulnerable to flooding and often exacerbated by subsidence (negative vertical land motion; VLM). The U.S. Environmental Protection Agency plays a key role in mitigating risks from ASTs. Satellite remote sensing provides a powerful tool to assess hazards and inform decision-making. Here, we present a roadmap for integrating remotely-sensed observations into decision-making frameworks. Using NASA observational products for end-users from remote sensing analysis (OPERA) VLM products derived from Sentinel-1, we map VLM at ~ 30 m resolution across Greater Houston–Galveston. Our analysis reveals widespread, spatially varying subsidence. We determine where VLM trends were linear from 2016 to 2023 and extrapolate them to estimate future VLM. Combining sea-level rise (SLR) scenarios with VLM data, we estimate that by 2050 ASTs in the region will experience at least 26.1 cm of relative SLR, with 10 (14.9%) exposed to more than 60 cm. Integrating a hydrodynamic model with spatially varying relative SLR shows that flooding hazards are amplified during a Hurricane Harvey-like event under future conditions. Overall, we demonstrate the importance of incorporating high-resolution VLM into hazard assessments to support decision-making.

**Supplementary Information:**

The online version contains supplementary material available at 10.1038/s41598-025-01970-8.

## Introduction

Coastal areas serve as critical hubs for industries reliant on large-scale transport and storage, including shipping, oil and gas, and manufacturing, where access to waterways facilitates efficient trade and logistics. Consequently, vital infrastructure, including facilities such as above-ground storage tanks storing petrochemicals, faces heightened vulnerability to natural and anthropogenic hazards linked to encroaching water. These include the increasing frequency of high-tide flooding^1^, event-driven risks from the convergence of elevated water levels, precipitation, and storm surge (e.g^2^.,), and structural degradation from saltwater intrusion (e.g^3^.,). Though often unaccounted in flood vulnerability analysis (e.g^4,5^.,), high resolution vertical land motion (VLM)—comprised of the sinking (subsidence) or rising (uplift) of the ground surface—can change elevation substantially over small spatial scales (~ hundreds of meters) due to various factors, such as fluid extraction and tectonic activity^6–9^. VLM, especially when coupled with sea level rise (SLR) and extreme events like storm surge or heavy precipitation (e.g., 2008 Hurricane Ike, 2017 Hurricane Harvey), can amplify hazards through enhanced flood exposure^10,11^, potentially triggering natural-technological accidents (Natech events; e.g^12^.,). Accurate and up-to-date monitoring of coastal infrastructure, integrating geospatial data on facility layers with VLM, SLR, and hazardous substances (particularly those that are reactive or ignitable), is essential for identifying vulnerabilities and guiding proactive protective measures.

The U.S. Environmental Protection Agency (U.S. EPA), whose mission is to protect human health and the environment, is tasked with preventing and responding to catastrophic spills, including those from facilities storing petrochemicals. These efforts are under the Oil Pollution Act, the Clean Water Act (CWA) and the CWA Hazardous Substance Facility Response Plan. To better enable preparedness and response efforts at national, state, and local levels, it is critical to understand the risk to vulnerable infrastructure (e.g^12,13^.,). Proactively identifying: (1) areas experiencing VLM and flooding, (2) infrastructure at risk of failure due to these hazards, and (3) stored substances of concern, provides vital, high resolution, time-sensitive information for responders and industry. Access to the most current and accessible data—from the national response center to local first responders—can improve time-critical decision-making to prevent system failures and improve responses to spill events, thus enhancing the protection of critical infrastructure and public health.

However, the scale of this challenge is substantial. For example, Anenberg and Kalman^14^ identified nearly 900 hazardous chemical facilities under U.S. EPA jurisdiction within 50 miles of the Gulf Coast; within 2.4 km (1.5 mi) of these chemical facilities are over 4 M people, 1700 + schools, and nearly 100 medical facilities. For such regulated facilities under the CWA Hazardous Substance Facility Response Plan, assessments must address potential impacts to public receptors, including schools, hospitals, homes, and drinking water sources. Integrating data on facilities subject to flood risk from extreme events and exacerbated by relative sea-level rise (RSLR; which includes VLM), alongside the risks posed by hazardous substances, is important for advancing public health protection. By addressing these interconnected risks, we may better equip facilities, communities and decision-makers to mitigate threats and respond to emergencies effectively.

As Houston, Texas, USA has one of the highest densities of above-ground storage tanks (ASTs) both nationally and globally, it serves as an ideal case study to examine the intersection of VLM, flooding and potential impacts from petrochemical releases. Additionally, Texas recently adopted a new regulation for ASTs (Title 30 Texas Administrative Code Chapter 338) aimed at improving the design, construction, operation, and maintenance of these tanks to protect groundwater and surface water supplies during accidents or natural disasters. With a population over 7.5 M, the Greater Houston region is the 5th largest metropolitan region in the United States^15^. In 2023 it generated $550B in GDP, and is home to the Port of Houston, one of the busiest ports in the world and a crucial hub of the global energy network^16–18^. A critical component of this network is the Houston Ship Channel, created by dredging natural waterways in the early 20th-century to transport goods throughout the greater Houston Region and Gulf of Mexico.

However, the Houston/Galveston region is highly vulnerable to flooding from precipitation events and storm surge—the Harris County Flood Control District estimates that a major flood occurs every two years^19^. This was particularly evident when Hurricane Harvey struck in 2017, causing damage exceeding $125B^20,21^. Such flooding impacts are expected to worsen due to RSLR, which is estimated to be 31 cm higher in the region by 2050 than today under the ‘intermediate low’ SLR scenario^10,22,23^, increasing intensity of tropical storms^24–26^, and local subsidence^27,28^. To reduce the impacts of flooding, the Harris County Flood Control District currently maintains 4000 km of channels, two major reservoirs, and a variety of other infrastructure. In response to Hurricane Harvey, they are investigating the feasibility of a $30B investment into the construction of 210 km of large (14 m diameter) underground tunnels that could drastically alleviate flooding hazards^29^.

VLM could impact the effectiveness of such current and future infrastructure and thus could be an important aspect of designing such protections. Long recognized as a regional hazard^30^, VLM prompted the establishment of the Harris-Galveston Subsidence District in 1975, a regulatory agency tasked with monitoring and managing groundwater extraction to mitigate subsidence (https://hgsubsidence.org/about/). Historically, the HGSD has relied on in-situ measurements from land-surveys and extensometers to measure VLM. In recent years, these methods have been complemented with observations from the highly accurate but spatially sparse Global Navigation Satellite System (GNSS) network and estimates derived from the satellite remote sensing technique of Interferometric Synthetic Aperture Radar (InSAR). InSAR confirms a dramatic reduction in subsidence since the 1990s^31^. Additionally, it reveals spatially variable ground motion—predominantly caused by aquifer compaction from fluid extraction^31–38^—that is not well captured in sparse in-situ observations alone. Other studies have assessed the impact of the changing land surface in the context of flood risk, both through event driven storm surge^39,40^ and through future projected SLR^27,35,39^. While these studies have identified key physical processes driving risk and potential vulnerabilities associated with RSLR, they have yet to be applied to actionable scenarios that inform infrastructure vulnerability and guide efforts to mitigate impacts on human health and the environment. This work addresses that gap by integrating satellite-derived products and analyses with assessments of infrastructure and impacts to facilities crucial to public health, a first step towards a robust decision-making framework.

To identify common remote sensing needs across the U.S. federal agencies, such as the EPA, the Satellite Needs Working Group (SNWG) under U.S. Group on Earth Observations meets biannually to interview federal agency end-users to identify products or solutions that address these needs^41^. The NASA Jet Propulsion Laboratory Observational Products for End-Users from Remote Sensing Analysis (OPERA) project (^42^; https://www.jpl.nasa.gov/go/opera/), which was formed as a SNWG solution, has been tasked with the development and production of operational analysis-ready surface displacement and VLM products for most of North America and all U.S. Territories derived from the European Copernicus Sentinel-1 satellite constellation and the upcoming NASA-ISRO SAR (NISAR) satellite. The OPERA algorithms are fully open source (https://github.com/opera-adt), its products are produced as new satellite data is acquired and are freely accessible through the NASA Distributed Active Archive Centers.

We estimate VLM at ~ 30 m resolution, using the methodology of refs 7 and 43 (see Methods), which detected linear rates of change with mm-scale accuracy. These estimates are comparable in accuracy to GNSS, but offer far broader spatial coverage, helping to resolve fine-scale spatial variability that GNSS alone may alias. Our VLM is derived from the standardized, ready-to-use OPERA displacement prototype, based on state-of-art time-series, rigorously validated InSAR algorithms (^43,44^). These products are being routinely produced and will be used for OPERA-VLM, the first operational VLM product over North America, providing a consistent and accessible baseline for a wide range of end users.

Here, we illustrate how VLM data in Greater Houston can enhance the monitoring and management of critical infrastructure in the context of natural and anthropogenic hazards combined with ongoing RSLR. We focus on designing a roadmap of how to use VLM information in planning, while accounting for uncertainties, to better understand future flood vulnerability of critical infrastructure. To the best of our knowledge, there is not a widely established roadmap leading from routinely estimated displacement to risk assessment of critical infrastructure. First, we present the overall picture of VLM as estimated using the OPERA-VLM prototype, placing it in context of prior work and demonstrating its use at critical infrastructure locations. Next, we discuss how temporal uncertainties should be used in conjunction with VLM rates to better estimate future land surface elevation. We then use a state-of-the-art flood model^45^ to illustrate how VLM could affect the future flooding hazard at ASTs during a Hurricane Harvey-like event. Finally, we discuss ongoing work aimed at integrating VLM and RSLR vulnerability with example scenarios of the ignitability and reactivity of hazardous substances that could potentially lead to Natech events. In short, we detail how OPERA-VLM can both refine our understanding of ongoing ground motion and support proactive management strategies for critical infrastructure in the face of accelerating RSLR.

## Results

### Linking VLM to current infrastructure vulnerability

**Fig. 1:**
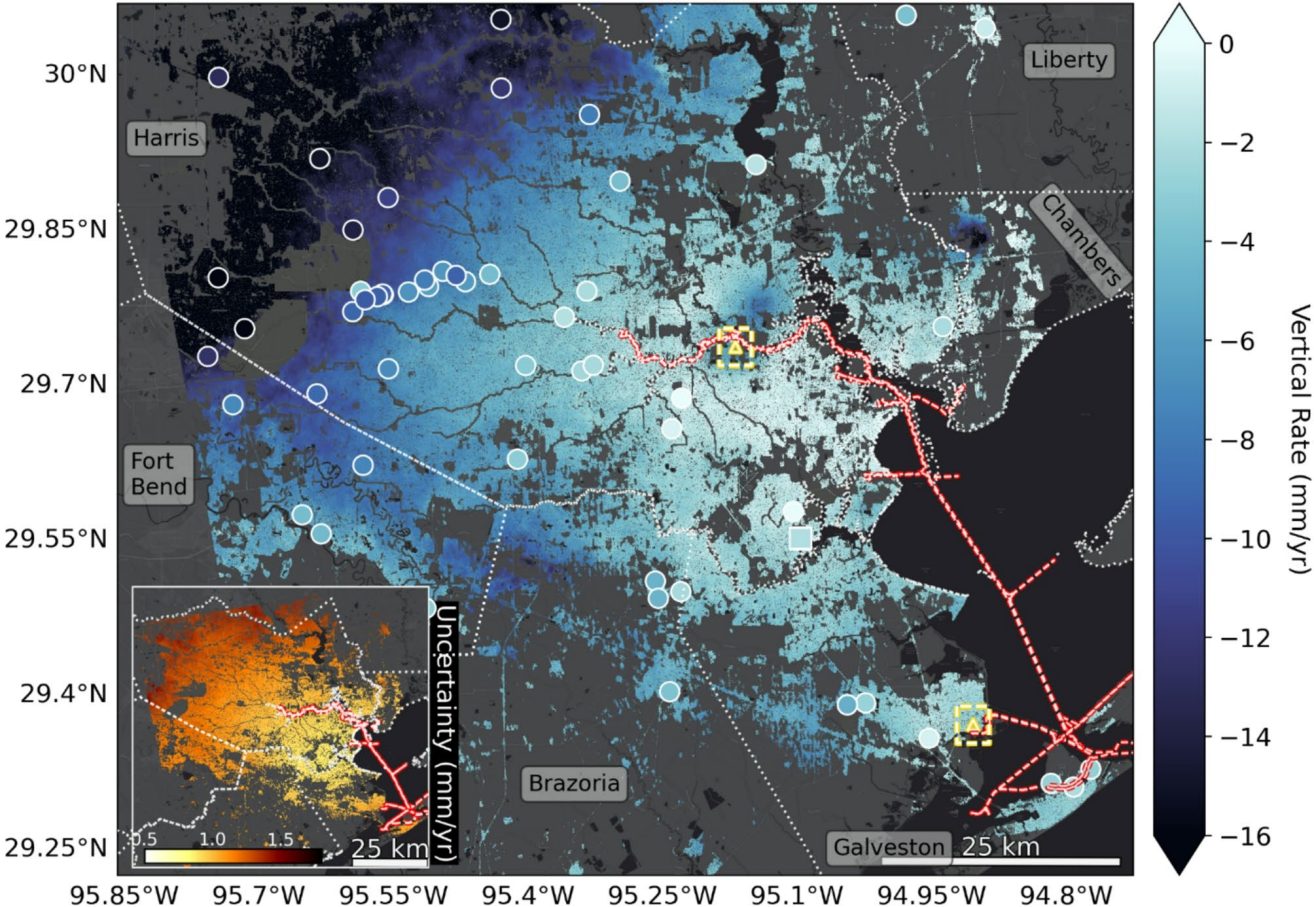
Vertical land motion rate map for the Sentinel-1 period spanning April 2016 to November 2023 in the ITRF14 reference frame. Markers show GNSS rates used in calibration (square) and validation (circular; accuracy assessment in Fig. S1). The corresponding VLM uncertainty map is shown in the inset map. Yellow triangles and surrounding rectangles indicate time series locations shown in Figs. 4, 5. The red/white dashed line marks the Houston Ship Channel, and white dotted outlines mark county borders.

Our OPERA VLM prototype rate map, referenced to the global navigation satellite system (GNSS) in the 2014 International Terrestrial Reference Frame^46^, spans April 2016 to November 2023 (~ 7.5 years) and reveals broad subsidence throughout the greater Houston/Galveston region (Fig. 1). We find that the city of Houston is subsiding at a median rate of − 5.3 ± 1.1 mm/year (+ /− 1 standard deviation), while Galveston is experiencing a slightly slower rate of − 3.1 ± 1.1 mm/yr. Across our study area, we find that 95% (~ 2-sigma range) of values fall between − 19.2 mm/yr and − 0.8 mm/yr. Overall, our VLM rates are well correlated with collocated, independent GNSS stations (RMSE of 1.2 mm/yr; Fig. S1); these stations estimate a median of − 4.3 ± 0.9 mm/yr, a rate of − 5.1 ± 0.9 mm/yr in Houston, and -2.9 ± 0.8 mm/yr in Galveston. VLM rate uncertainties are estimated via least squares with propagation of error of GNSS uncertainty resulting in the 99.7% range (~ 3 standard deviations) of 0.9 to 1.4 mm/yr (Fig. 1 inset). 96.6% of pixel locations are significant at 1-sigma. These uncertainties are crucial for correct interpretation of the OPERA VLM and should be used in conjunction with the VLM to provide a realistic range of the contemporary rate.

Across our study area there is considerable spatial variability, with subsidence rates generally increasing from effectively 0 mm/yr in La Porte in southeast Harris County (Fig. S2; − 0.8 ± 1.1 mm/yr), to < − 20 mm/yr in northwest Harris County. Subsidence in northwest Harris County has historically been attributed to groundwater extraction for industrial and municipal use^47^. The decline in ground water levels continues to be a concern given water demands^48^, especially with the extended drought and population increase. Most of the water is extracted from the Chicot and Evangeline aquifers which dips and thicken from the northwest to the southeast^49,50^, as reflected in both changes in groundwater level and the observed radial pattern of subsidence observed here and previously (e.g^31,51^.,). We note additional hotspots of subsidence near Mont Belvieu, Channelview, and across the border of Fort Bend and Brazoria County. At Mont Belvieu, subsidence has been attributed to hydrocarbon extraction at the Barber’s Hill Oil Field^31^. The subsidence at Channelview, which traverses the Houston Ship Channel, does not display a clear relationship with nearby groundwater extraction^38^ but is located adjacent to a salt dome^31^ which could cause localized VLM. Salt domes, formed as buried salt layers rise through overlying rock due to pressure and buoyancy, are often associated with hydrocarbon production and underground storage, creating complex patterns of VLM^30^. Additionally, hydrocarbon extraction along aseismic faults that exhibit slow horizontal creep (< 2 cm/yr) have been implicated in localized VLM^34^. Such activity likely contributes to the band of subsidence near Fresno (Fort Bend County, 5 km south of where it borders the intersection of Harris and Brazoria County), part of which coincides with the Arcola Fault^38,52^.

**Fig. 2:**
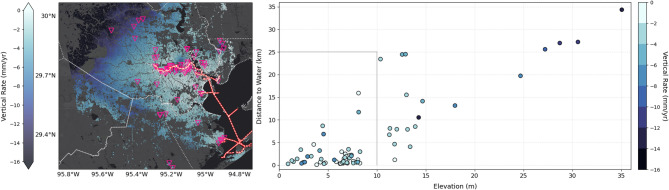
Above-ground storage tank (AST) vulnerability. (left) Map showing the 73 ASTs facilities considered in this study overlaid on Fig. 1. (right) Scatter plot showing facilities as a function of elevation and distance to water as a proxy for vulnerability to flooding. The cluster (within gray outline) of infrastructure at low elevation nearby water, are most vulnerable today. Those with higher subsidence (darker blue), especially in this cluster, are more likely to become vulnerable in the future. Note the size of color bin reflects the typical uncertainty of ~ 1 mm/yr.

Such localized VLM data can be used to more effectively identify coastal infrastructure vulnerable to the impacts of RSLR, thus facilitating better-informed resource allocation and more targeted adaptation planning. As indicated earlier, the Houston Ship Channel is one of the world’s largest petrochemical complexes with ASTs storing petroleum products and hazardous substances (Fig. 1, red/white line). ASTs are the most vulnerable component of industrial facilities in storm events and result in the largest oil and chemical spills during extreme weather events, which have been increasing in intensity^53^. Some ASTs have not been adequately designed to withstand increasing extreme weather events over long periods of time (e.g^54^.,). Flood inundation depth is a critical parameter controlling AST vulnerability (e.g^55^.,), as it determines the likelihood of petrochemical reactivity and ignitability when floodwaters encounter stored substances, potentially triggering Natech events^56^. We address inundation by estimating the risk of current and future flooding for 73 critical storage facilities in our study area by analyzing their proximity to coastal waters or the Ship Channel (Fig. 2). Our analysis reveals that 53 facilities (72.6%) are located both within 25 km of these water bodies—a distance potentially affected by storm surge (e.g^57^.,)—and at elevations below 10 m, placing them at enhanced risk of flooding. Of these, six are located on ground less than 3 m elevation and within 1 km from water, and could be considered most at risk from flooding. Such vulnerability will be exacerbated by VLM: half of the 53 exposed stations are sinking faster than − 2.2 ± 1.0 mm/yr and 2 of the 6 most at risk are subsiding at or faster than − 6.0 ± 0.1 mm/yr. While not a rigorous vulnerability analysis, this basic methodology is broadly applicable to a range of infrastructure and can be used to quickly identify at-risk infrastructure and help prioritize management strategies.

### Considerations for quantifying future VLM

Until about 2050, future U.S. SLR scenarios remain fairly consistent across emissions pathways^58,59^, creating an opportunity to design and implement protective actions now that yield long-term economic and environmental benefits (e.g^60–62^.,). However estimates of future VLM from these scenarios are conducted at tide gauge locations^63^, thus lacking the spatial resolution and coverage needed for critical infrastructure monitoring. Others have assumed that VLM behaves linearly and extrapolated observed rates into the future^27^. However, VLM can behave nonlinearly^6,28,64^, making it unsuitable for linear extrapolation. Thus, both high spatial resolution and robust understanding of linearity are needed to inform management decisions by the EPA, as well as state and local governments, not only for petrochemical infrastructure but also for other essential sectors.

**Fig. 3:**
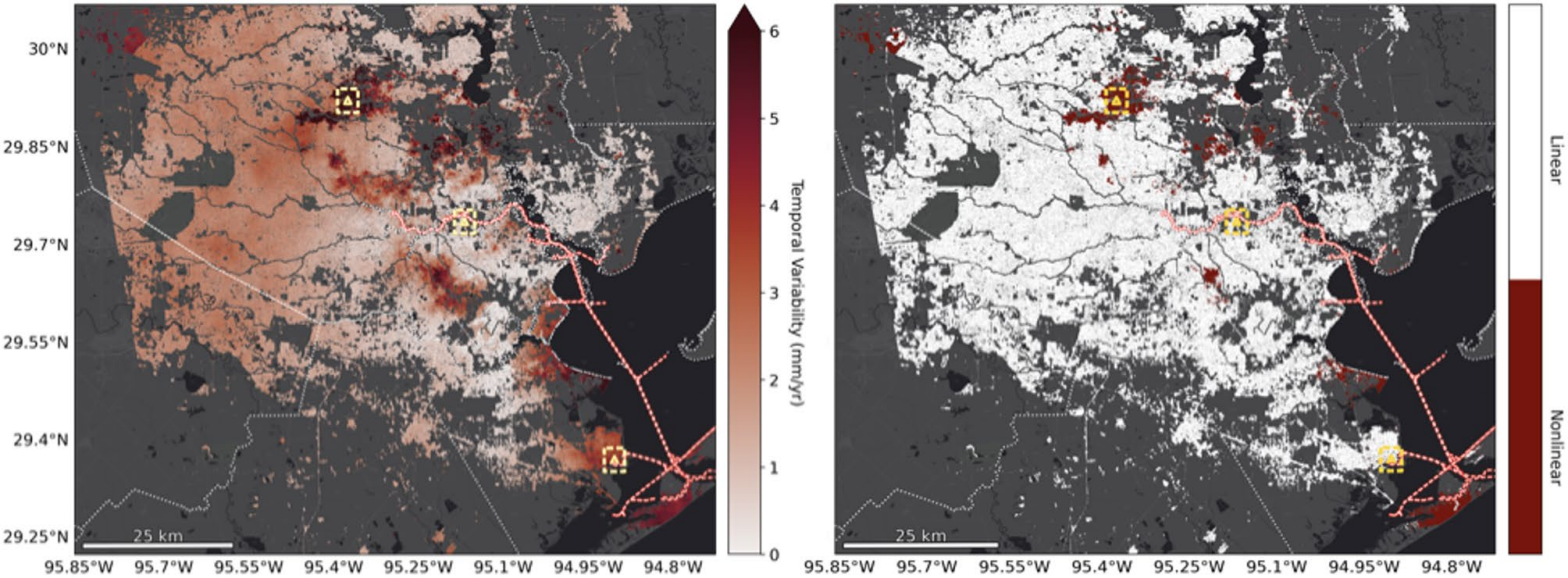
Vertical rate Temporal Uncertainty, a proxy for nonlinearity. (Left) temporal variability estimated by comparing the linear rate over the full record with that computed over smaller subsets. (Right) mask showing linear/nonlinear based on the 2-sigma range (4.4 mm/yr) of the temporal variability.

Here, we examine the assumption of linearity in our time series by estimating temporal variability, a metric of nonlinearity, which compares the agreement between a linear trend computed over the full record length with trends computed over shorter records (> 3 years; 28). Over greater Houston/Galveston, we find a median temporal variability of 1.9 mm/yr, with 95% of values between 0.3 and 5.1 mm/yr (Fig. 3a). Following ref^28^. we use this temporal variability to create a binary mask that marks pixels above (below) the 2-sigma range of 4.4 mm/yr as nonlinear (linear; Fig. 3b). Therefore 95.8% of locations behave linearly within the observation period (April 2016 to November 2023), including 71 of 73 ASTs.

Nonlinear VLM is predominantly observed in northwest and west Houston, driven primarily by anthropogenic groundwater extraction, which fluctuates in response to societal demands and policy changes. Importantly, our assumption of linearity is limited to the analysis time frame and so may not accurately reflect conditions prior to, or beyond, this period. This masking approach serves as a useful screening tool to quickly identify areas where linear extrapolation of VLM may inform RSLR resilience strategies. While straightforward and readily implementable, it should be complemented by further investigation into the underlying physical processes causing deformation whenever possible.

**Fig. 4:**
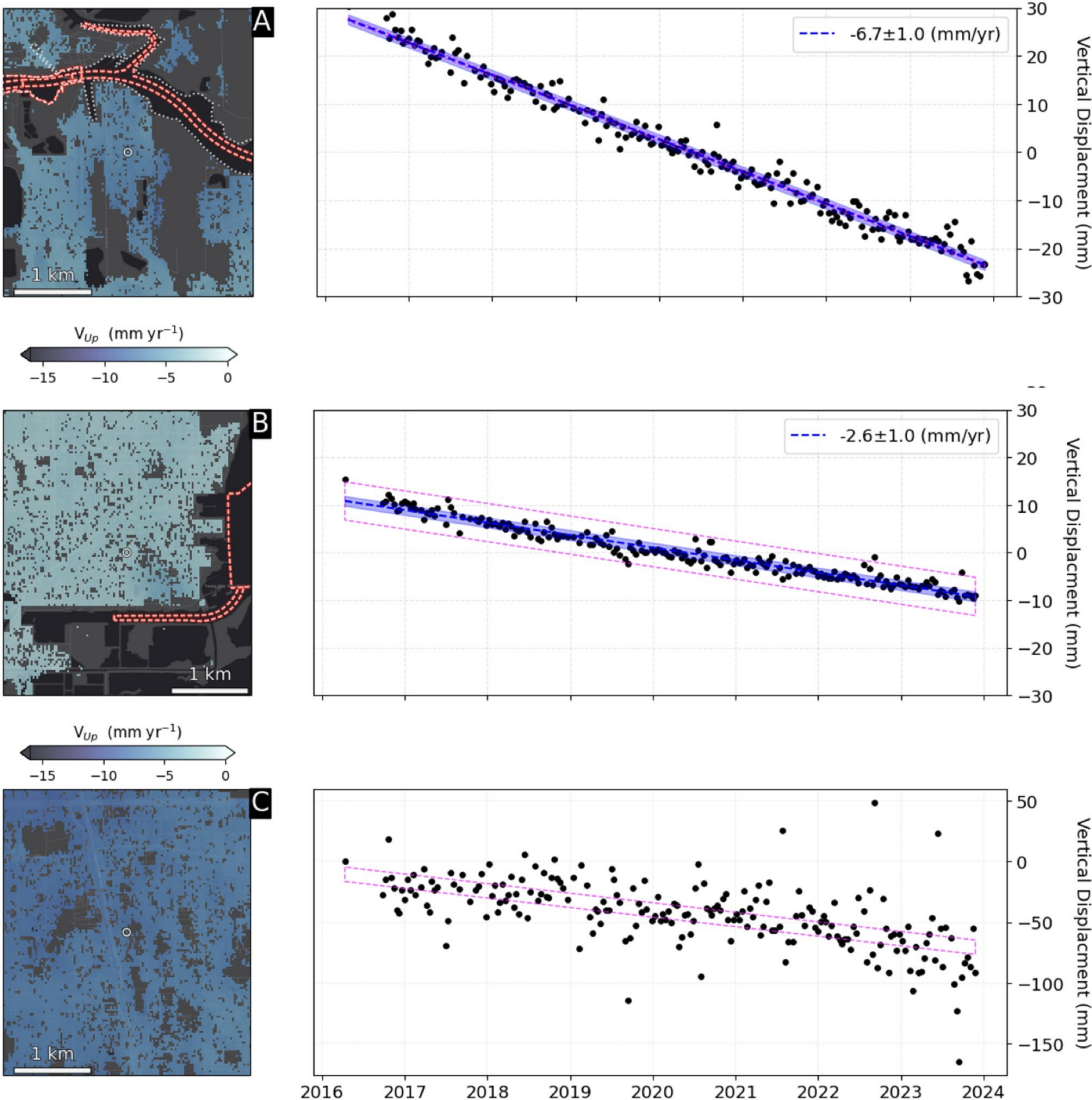
Vertical displacement time series at (**a**) Buffalo Bayou and (**b**) Texas City, Galveston and (**c**) North Houston (near Magnolia Gardens). Blue shading represents the time-series uncertainty, while the magenta dashed line shows the uncertainty due to temporal variability. Buffalo Bayou has a low temporal variability of 0.3 mm/yr, while Texas City is relatively high (4.0 mm/yr) but remains linear. North Houston displaying nonlinearity—especially after about 2021—that is correctly captured by the nonlinearity indicator (temporal variability of 5.9 mm/yr). Note different y axis on (**c**).

To further demonstrate temporal linearity, we highlight two coastal locations near critical infrastructure that exhibit low and high temporal variability (Fig. 4). First we highlight a section of Buffalo Bayou, just south of the Ship Channel in northern Pasadena, where numerous oil refineries occupy lands built on former wetlands. Here, we observe rapid subsidence of − 6.7 ± 1.0 mm/yr that is clearly linear during our period of analysis (2016–2023), and confirmed by a low temporal variability estimate of 0.4 mm/yr (Fig. 4a). Texas City, abutting western Galveston Bay, displays a relatively high temporal variability of 4.0 mm/yr but is also linear: a linear model (− 2.6 ± 1.0 mm/yr) explains most of the variance of the timeseries (r^2^ = 0.94; Fig. 4b) supporting the 2-sigma range (4.4 mm/yr) threshold adopted for classifying linearity. Conversely, Fig. 4c shows a nonlinear (temporal variability of 5.9 mm/yr) time series at Magnolia Garden, which displays a relative acceleration of subsidence starting in 2022.

**Fig. 5:**
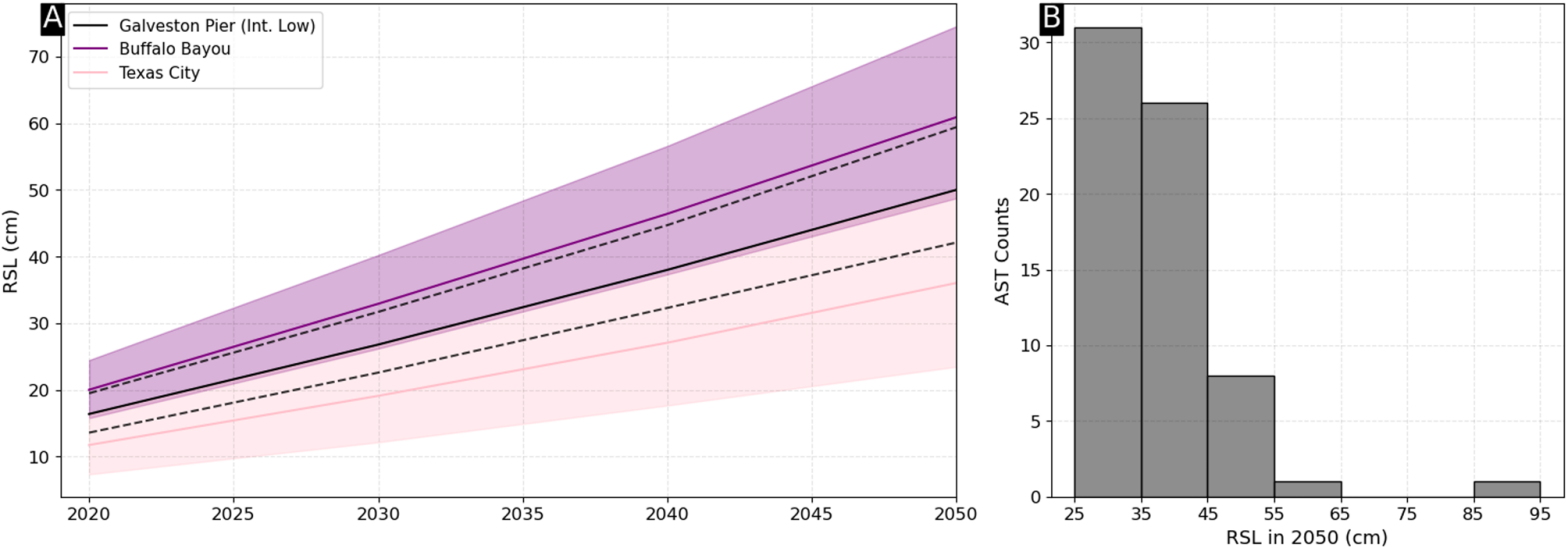
RSLR extrapolations (left) Example time series Galveston Pier 21 shows the RSLR estimate from the U.S. Intermediate-Low SLR scenario, containing a VLM estimate of − 5.1 ± 0.1 mm/yr^59^. Buffalo Bayou and Texas City use OPERA-VLM rates of − 6.7 ± 1.0 mm/yr and − 2.6 ± 1.0 mm/yr, respectively. (Right) RSL at 67 above-ground storage tanks (within 25 km of water) in 2050 using OPERA-VLM, emphasizing the impact of spatial variability of VLM.

The two locations highlighted in Fig. 4further emphasize the variability in subsidence across Houston, which as previously noted ranges from − 19.3 mm/yr to − 0.8 mm/y (~ 2-sigma range). Accurately capturing this range is critical for estimating future RSLR and assessing associated vulnerability. Currently, the U.S. SLR scenarios apply a single VLM estimate of -5.1 ± 0.01 mm/yr, which underestimates uncertainty and does not reflect the full spatial variability of subsidence. To better account for this variability, we use our InSAR-derived OPERA VLM rates—which are predominantly linear according to our temporal variability metric—to improve RSLR projections. Building on ref^28^., we replace the VLM component in the U.S. SLR scenario framework with our OPERA VLM rates and employ a probabilistic approach to estimate confidence intervals for RSLR projections through 2050 (see Methods). In the northern Pasadena location, RSL is projected to be an additional 7.2 cm higher due to VLM not captured by the U.S. SLR scenario estimates, a 14.3% increase (Fig. 5a). Conversely in Texas City, the projected RSL is 22.6% lower than the intermediate-low SLR scenario. Overall, 58.2% of the land area is subsiding at rates exceeding the SLR scenario (− 5.1 ± 0.1 mm/yr). Within 25 km of the Ship Channel and/or the coast, 26.9% of the land is subsiding at rates faster than − 5.1 mm/yr, and ten ASTs (14.9%) are sinking at rates exceeding − 5.1 mm/yr (Fig. 5b), emphasizing that areas relatively safe today could be at risk in the future. Conversely, some currently at-risk areas may face less severe hazards than previously anticipated, underscoring the need for strategic allocation of resources.

### Assessing flood risk at critical infrastructure

Finally, we explore how the inclusion of RSLR may alter the range and severity of extreme flood hazards. Specifically, we use an existing state-of-the-art flood model to simulate a Hurricane Harvey-like event^45^. We avoid the common use of simplified ‘bathtub models’^27,65^ to simulate inundation, given that Sanders et al.^66^, recently highlighted their inaccuracies. Incorporating RSLR with spatially varying OPERA VLM, we find that six (9.2%) of the ASTs experience a significant increase in flooding (|Δ|> 10 cm; see Methods), and none experience significantly less flooding. We find that two locations adjacent to the Houston Ship Channel experience flooding greater than 80 cm, suggesting that these are the most at risk in 2050 from a Hurricane Harvey-like extreme event. Importantly, the remaining 34 ASTs showed no change in flooding. Overall, 12% of our study area coincident with the model domain experienced a significant change in flood depth (Fig. S3). Flood depth increased at 85% of locations, most notably along the Houston Ship Channel and the reservoirs in western Harris County. While here we focused on the tail end of hazardous events, it is important to note that RSLR can also alter flooding associated with daily tides and smaller, more frequent storms, potentially changing the risk of additional ASTs. For example, more frequent, albeit smaller inundation could lead to degradation of facilities while changes in the VLM could alter runoff flow paths, leading to precipitation induced flooding inland from water bodies. Such possibilities underscore the need for incorporation of high resolution VLM into physics-based flood inundation modeling.

## Discussion

**Fig. 6:**
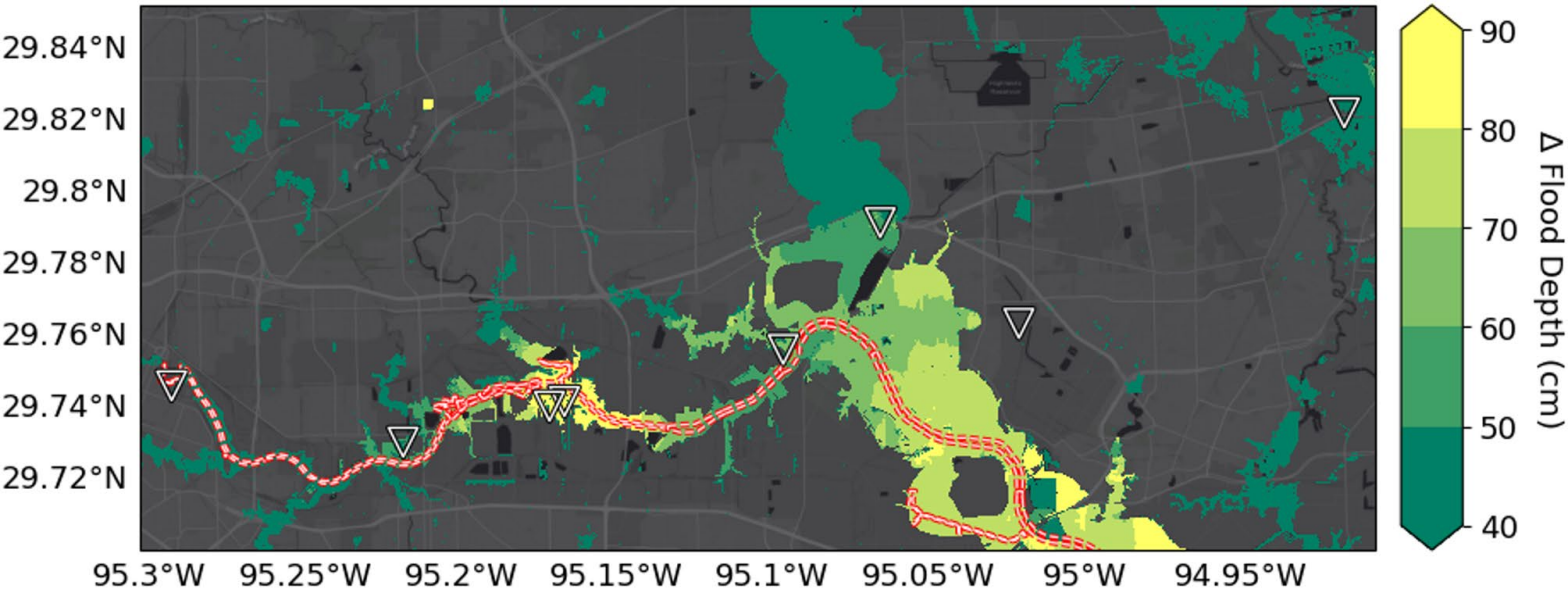
Impact of RSLR on future flooding from Hurricane Harvey-like event. Higher values indicate a greater depth of simulated flooding in 2050 (due to RSLR) relative to Hurricane Harvey alone. Triangles mark the 6 AST facilities in our study area that experienced significant changes, and the red/white dashed line again marks the Houston Shipping Channel.

The roadmap presented here is a first step towards incorporation of RSLR into robust decision-making frameworks. With the OPERA VLM prototype, we show that Houston is subsiding rapidly, but at different rates throughout the city, potentially worsening exposure to flooding at locations of importance to national security (Figs. 1, 2). Next, we illustrate a technique for diagnosing linearity, finding that subsidence is primarily linear from April 2016 through November 2023 (Figs. 3, 4), which allows future extrapolation of the rate (Fig. 5a). Using the ASTs as an example of critical infrastructure susceptible to flooding, we showcase a method to combine VLM with future SLR scenarios, finding that ASTs will be exposed to RSLR of least 26.1 cm in 2050, while 10 (14.9%) may experience RSLR of 50 cm or more (Fig. 5b). Finally, we show that, if possible, a state-of-the-art hydrodynamic model should be used with spatially varying RSLR to fully understand the flood hazard; incorporation of RSLR caused substantially more flooding at specific ASTs when a Hurricane Harvey-like event occurred under a future RSLR scenario (Fig. 6).

However, we caution that our roadmap is a prototype, a first step that does not capture the full range of hazards that infrastructure, like ASTs, could be exposed to, such as saltwater intrusion (e.g^67^.,), more frequent shallow inundation (e.g^1^.,), changing tidal ranges (e.g^68^.,), groundwater inundation (e.g^69^.,), and more. While our uncertainty estimates account for noise in InSAR processing, inaccuracies in digital elevation models, and assumptions in flood modeling (see *Methods*), there are nevertheless uncertainties in future land and water use changes, infrastructure development, and evolving coastal dynamics that will require ongoing monitoring and analysis as new information becomes available. The OPERA VLM product, especially when created with data from the upcoming NISAR mission, will be uniquely situated to meet this challenge in North America—combining state-of-the-art open source InSAR processing algorithms processing with cloud capabilities. Such developments enable low-latency data updates that have hitherto been impossible due to the complexity and computational intensity of InSAR processing (Meyer et al., 2025). Ongoing monitoring is increasingly important as the probability of extreme events, including with Harvey-like rainfall, increases in the future^70^. Indeed, Texas is the leading state in both the frequency and costs of billion-dollar weather and climate disasters (NOAA National Centers for Environmental Information, U.S. Billion-Dollar Weather and Climate Disasters (2024).

Particularly damaging is the effect of extreme events on petrochemical facilities, especially given that 89 million people in the U.S. live or work within ~ 3.2 km of a high-risk chemical facility (cisa.gov/cfats). For example, flood damage to ASTs during Hurricane Harvey caused the release of at least 4.6 million pounds of chemical pollutants and damage to at least 14 toxic waste sites^71^ and water reactions with petrochemicals are a leading cause of Natech events^13,56^. Indeed, the U.S. Chemical Safety Board specifically recommended incorporating flood maps as process safety information and addressing extreme weather vulnerabilities and critical safeguards (https://www.csb.gov/assets/1/6/biolab_investigation_report_2023-4-24.pdf). The EPA is currently developing a tool that integrates the locations and chemical contents of petrochemical facilities—including water-reactive substances—with public health and environmental data, such as drinking water supplies, public facilities (e.g., homes, schools, hospitals), and fish and wildlife habitats (Fig. S4). As high-resolution OPERA VLM data, combined with SLR, is incorporated into the tool on a national scale, it will enhance the ability to make strategic decisions for disaster prevention and response. The prototype roadmap presented here illustrates how end users can leverage ready-to-analyze satellite information from the OPERA project alongside future scenarios to better understand and mitigate coastal hazards. As OPERA VLM products become available over North America, this framework can serve as a model for mitigating risks in other vulnerable areas of the continent.

## Methods

### OPERA vertical land motion

We obtained 206 Sentinel-1 Single Look Complex (SLC) images along ascending track 34 spanning April 12, 2016, to November 26, 2023, from the Alaska Satellite Facility. The methods used for estimating VLM are nearly identical to those in^7^, with key points reproduced here for completion. We prepared the SLC images for time series analysis by coregistering them to a single geometry using the ISCE-2 sentinelStack workflow^72^. Topographic effects are accounted for using the Copernicus GLO30 DEM. We use the InSAR time series processing software FRInGE^73,74^, a prototype of the OPERA production algorithm Dolphin^43^, to generate full-resolution (approximately 2.3 m in range, 15.6 m in azimuth) wrapped interferograms. FRInGE uses both a SqueeSAR-like^75^ strategy for identifying distributed pixels while using the traditional amplitude dispersion method to identify persistent scatters^76,77^. We identify neighborhoods of statistically self-similar distributed scatters using a window size of 33 pixels in range and 15 lines in azimuth, and an amplitude dispersion threshold of 0.4 for determining PS pixels. FRInGE uses every possible interferometric pair from the given SAR network to extract the maximum amount of phase information. To improve efficiency, the full time series is split into mini-stacks of 15 SAR images each, for which the covariance matrix is estimated for each neighborhood of pixels^78^. The covariance matrix is then decomposed into eigenvectors and corresponding eigenvalues. The phase of the largest eigenvector is used to generate the wrapped phase time series with reduced temporal decorrelation effects.

We multilook the resulting 206 wrapped interferograms by a factor of 5 × 2 in range and azimuth, respectively, resulting in ~ 25 m pixel size. We assume there are no large displacements across water bodies, so we mask and interpolate using inverse distance weighting across them to minimize phase jumps during unwrapping with Snaphu^79^. The single reference unwrapped time-series phase is converted to displacement by scaling with a factor of -λ/4π, where λ = 5.6 cm, the Sentinel-1 wavelength. We estimate line-of-sight (LoS) velocities by fitting a linear trend plus sinusoid with an annual and component to the displacement time series. The LoS velocity uncertainty is the standard deviation of the regression slope^80^. Temporal variability is estimated by comparing the best fit trends to data from different moving windows with the trend estimate from the full data record. We use the median absolute deviation of the disagreement between the moving window trend (from 3 to 5 years in length) and full-record trend to quantify temporal variability; see^28^ for full details. We geocode our results to a posting of approximately 30 m × 30 m.

We apply geophysical and geometrical corrections using MintPy^80,81^. We first correct for atmospheric effects using ERA5 reanalysis with PyAPS^82^, solid earth tides with PySolid^83^ and topographic residuals^84^. We then remove long-wavelength horizontal motion due to plate tectonics using the model for the North American plate^85^. We remove outliers in the time-series by dropping SAR acquisitions outside of the 95% confidence interval, as determined relative to the Median Absolute Deviation of residual tropospheric delay^81^. We reduce residual noise using empirical orthogonal function analysis (e.g^86^.,) by decomposing the time series into orthogonal eigenvectors and reconstructing it using only the leading 5 vectors which contain 88.3% of the variance. Note that we remove an annual cycle from the time series by fitting a sinusoid prior to decomposition.

The total effect of applied corrections is small: averaged over the study area, velocities are 0.6 mm/yr lower than before the correction, and 95% of residual velocities are between − 1.2 mm/yr and 0.8 mm/yr, on par with average uncertainty of 1.1 mm/yr. Uncertainty is reduced on average by 0.2 mm/yr with a maximum reduction (pixel-wise) of 0.6 mm/yr. This is expected given the small size and flat terrain of the study area relative to the long wavelength spatially correlated tropospheric noise^87^ and coarse resolution of the ERA5 weather model^88^.

We only consider coherent pixels (temporal coherence greater than 0.8) and those on land according to the water mask available from Open Street Maps^89^.

We leverage the Global Navigation Satellite System (GNSS) network to tie our relative InSAR LoS velocity into the ITR14 terrestrial reference frame^46^. There are 55 GNSS stations in our study area available from the Nevada Geodetic Lab^90^with velocity and uncertainty estimates that are consistent in space and time with our InSAR rates (2016–2023). Due to limited constraints in decomposing satellite LoS with only one viewing SAR geometry (descending track 41 does not cover coastal Houston/Galveston), we projected our corrected LoS displacements to the vertical by assuming horizontal motion to be negligible. We take this assumption to be justified in this case as we removed known contributors to large-scale horizontal land motion, namely solid earth tides and rigid plate motion, and the gradient of horizontal motion due GIA is negligible (< 0.03 mm/yr^91^;). We obtained vertical rates using the equation:

1 $$V_{Up } = \frac{{V_{LOS} }}{cos \left( \theta \right)}_{{}}$$

where θ is the local incidence angle. We tie our map to the GNSS station at NASA by adding GNSS velocity (2015–2023) to InSAR rates (2016–2023) and propagating the uncertainty to obtain our final velocity and uncertainty estimates^92^.

We empirically minimize local spatially correlated noise in the time series (Fig. 4a,b) by first computing the Pearson correlation coefficient (⍴) between the detrended pixel timeseries of interest and all pixels within a 2.5 km radius. We then compute the spatial average of pixels with ⍴ > = 0.95 and remove it from the original time series of interest and recompute the trend and uncertainty using bootstrapping. Resulting trends are not sensitive to either the length of the radius or the correlation threshold. To further minimize local scattering, geolocation, and atmospheric errors, we average within a 2-pixel radius. Similarly, we estimate VLM at above-ground storage tank facilities using an average of VLM within 1 pixel distance of the facilities; results are insensitive to alternative pixel distances.

We utilize SLR scenarios from the U.S. Interagency Technical Report on Sea Level Rise^10^ which are derived from the IPCC 6th Assessment Report^23^. We focus on VLM contributions to scenarios in the near-term, by 2050, at a point where scenarios do not differ significantly^10^. Specifically, we use the intermediate-low scenario, a medium pathway of future emissions which agrees well with extrapolated observations. As in^28^, we replace the VLM component used in the underlying framework^63^ with our OPERA VLM estimates. We calculate the confidence interval for contemporary estimates using the z-score in a normal distribution to convert mean VLM rates and their uncertainties (standard deviations) to the 17th and 83rd percentiles as reported in the IPCC.

### Flood modeling

For estimating elevation of ASTs, we used a DEM derived from “Upper Coast LiDAR” data acquired in 2018 as Texas Strategic Mapping Program (StratMap), titled “Upper Coast Lidar”. This dataset has a horizontal accuracy of ± 20 cm and vertical RMSE ≤ 10 cm^93^. This DEM was also integrated into the flood simulation of Hurricane Harvey-like storm executed using the Parallel Raster Inundation Model (PRIMo), a sophisticated mechanistic flood inundation forecasting framework^45^. To integrate future RSLR, we modify the digital elevation model (DEM) values by extrapolating VLM rates to 2050 and adding them to the DEM. We do not consider the linearity/nonlinearity of the VLM time series and interpolate to fill gaps due to loss of temporal coherence. We therefore focus our analysis on the significant above-ground storage tank facility locations, where VLM behaves linearly, and temporal coherence is above our masking threshold of 0.8. We increase the ocean boundary condition by 0.27 m, the ocean contribution to SLR in 2050 under the Intermediate-low SLR scenario. We consider a flood difference of greater than 10 cm absolute significant based on the vertical accuracy of ~ 10 cm. The full model details can be found in^45^.

## Electronic supplementary material

Below is the link to the electronic supplementary material.

Supplementary Material 1

## Data Availability

All data needed to evaluate the conclusions in the paper are present in the paper and/or the Supplementary Materials. InSAR processing software is freely available on GitHub and archived on Zenodo. ISCE-2 is at https://github.com/isce-framework/isce2 and https://zenodo.org/record/8157051, FRInGE is at https://github.com/isce-framework/fringe/tree/main, and https://zenodo.org/record/8157065, and MintPy is at https://github.com/insarlab/MintPy and https://zenodo.org/record/7502839, GNSS data and MIDAS rates and uncertainties are available from the Nevada Geodetic Lab at http://geodesy.unr.edu. Sentinel-1 single look complex images (SLC) are available at the Alaska Satellite Facility Distributed Active Archive Center (https://asf.alaska.edu/). The rate and associated uncertainty map produced in this work are available in the supplementary files in both geotiff and kmz (Google Earth) format. The historical flood depth model prediction is available at https://datadryad.org/stash/dataset/doi:10.7280/D1NX1W (H10.tif), and the future flood depth model prediction is available in the supplementary files in geotiff format.
