# Supplementary material for: Satellite-based vertical land motion for infrastructure monitoring: a prototype roadmap in Greater Houston, Texas
Source: Sci Rep. 2025 May 16;15:17041. doi: 10.1038/s41598-025-01970-8 (PMC12084606; doi:10.1038/s41598-025-01970-8)
Supplement: Supplementary file 1 — Supplementary Material 1 [file 41598_2025_1970_MOESM1_ESM.docx]

# Supplementary Information to: Satellite-based vertical land motion for infrastructure monitoring: A prototype roadmap in Greater Houston, Texas

**B. Buzzanga^1,2*^, M. Govorcin^1^ , F. Kremer^3^, J.E. Schubert^8^, D.P.S. Bekaert^1^, B. Schaeffer^4^, P. Milillo^5,6,7^, A.J. Williams^4^, B.F. Sanders^8^, A.L. Handwerger^1,2^, S. Staniewicz^1^**

1. Jet Propulsion Laboratory, California Institute of Technology, Pasadena, CA, USA
2. Joint Institute for Regional Earth System Science and Engineering, University of California Los Angeles, Los Angeles, California, USA
3. U.S. Environmental Protection Agency, Office of Research and Development, Cincinnati, OH, USA
4. U.S. Environmental Protection Agency, Office of Research and Development, Research Triangle Park, NC, USA
5. Department of Civil and Environmental Engineering, University of Houston, Houston, TX, USA
6. Department of Earth and Atmospheric Science, University of Houston, Houston, TX, USA
7. Microwaves and Radar Institute, German Aerospace Center (DLR), Oberfaffenhofen, Germany
8. Department of Civil and Environmental Engineering, University of California, Irvine, CA, USA

*Corresponding Author Email: buzzanga@jpl.nasa.gov;

© 2025. All rights reserved


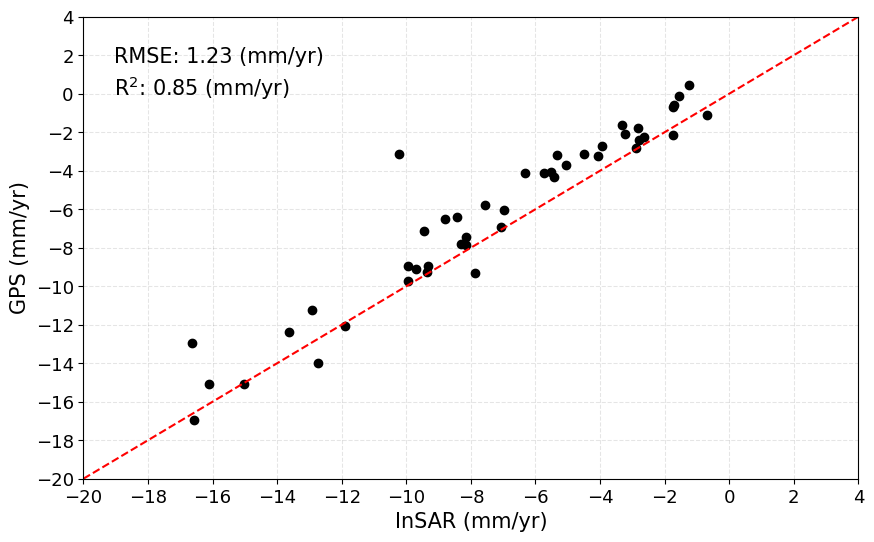


**Fig. S1** Comparison of independent GNSS rates with colocated InSAR rates within a 1-pixel, a measure of InSAR accuracy.


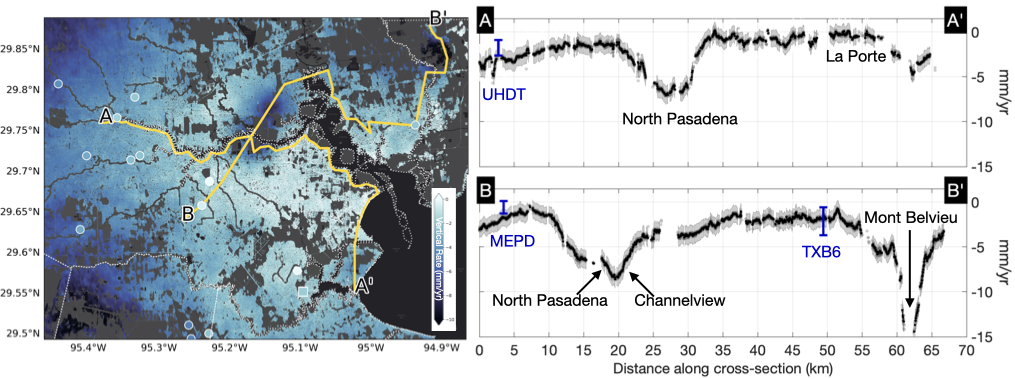


**Fig. S2** Transects along Houston Ship Channel (A-A’) and through the central/northeastern part of the study area (B-B’) showcasing spatial variability and hotspots of subsidence described in the results section.


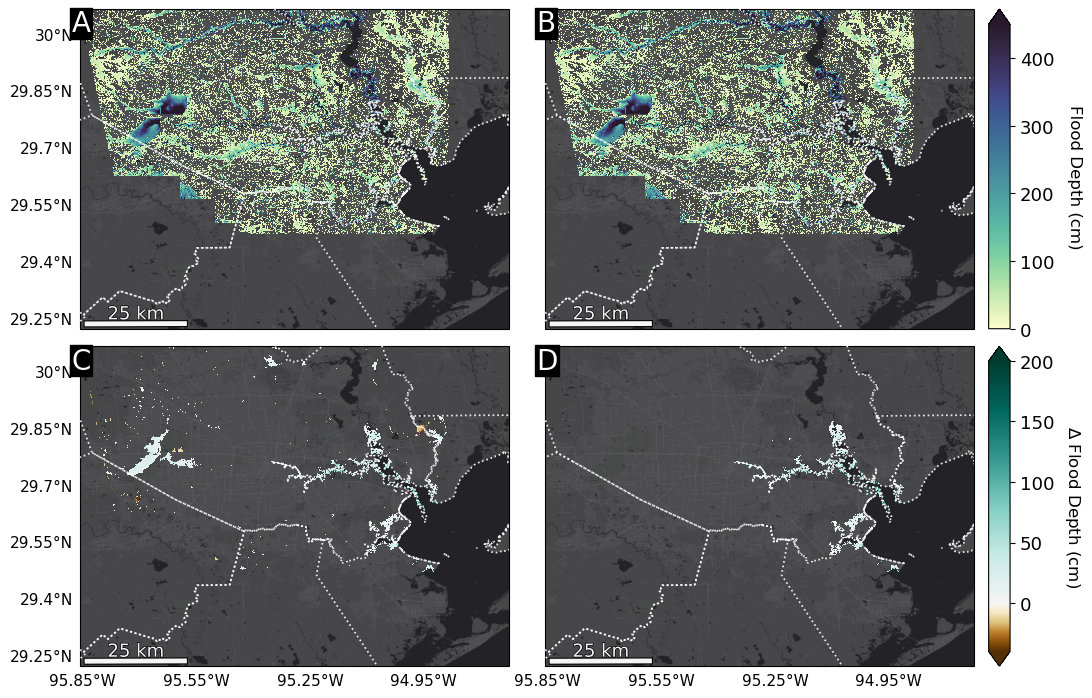


**Fig S3. Flooding due to Hurricane Harvey-like event.** a) flood depth resulting from hindcast (44) b) flood depth with spatially varying extrapolated OPERA VLM and the intermediate-low SLR scenario in 2050 c) Depth difference in 2050 due to relative SLR with OPERA VLM (panel b-a). d) Depth difference in 2050 due to relative SLR with spatially uniform VLM from U.S. SLR scenario.


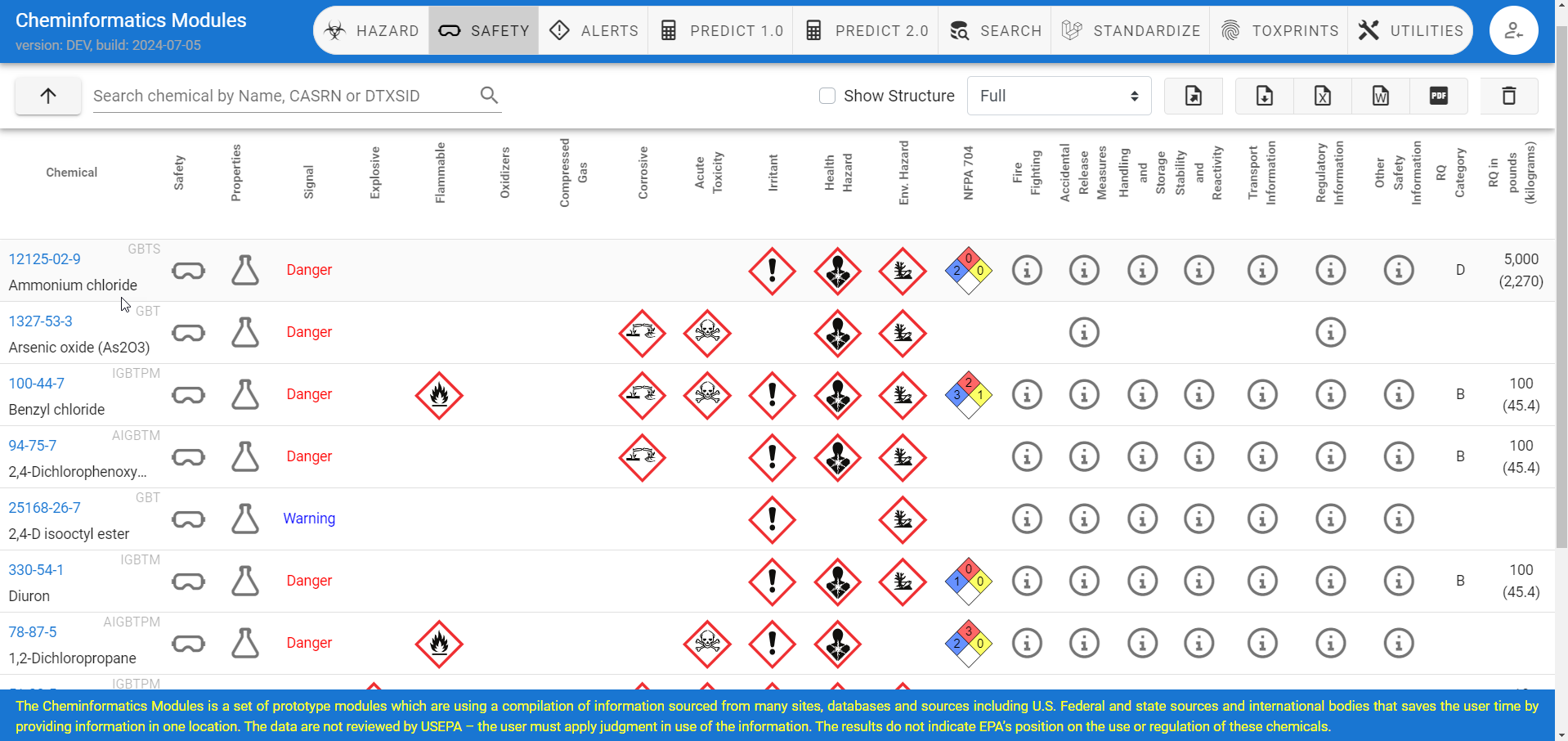


**Fig. S4. The Cheminformatics Modules Safety Profile for the Clean Water Act Hazardous Substances,** chemicals (https://hcd.rtpnc.epa.gov/) as displayed in the Safety Module. Each of the informational icons (
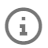
) shown in the safety profile can be clicked to show the underlying data. It contains the critical information for emergency responders and for industry to consider physical, chemical, and biological effects of the parent compound and by-products. For example, for acetic anhydride clicking on the “Stability and Reactivity” informational icon provides details in a new window and indicates that the chemical is flammable, reacts violently with water, and the reaction is influenced by the presence of acids. Alternatively, a substance such as potassium cyanide reacts with water creating hydrogen cyanide, a highly toxic chemical that can result in fatalities. These types of reactions can lead to Natech events. Identifying these substances in advance, at specific facilities, and integrating the VLM/RSLR data on potential inundation will prevent releases and significantly assist in emergency response.
